# Attention and schema violations of real world scenes differentially modulate time perception

**DOI:** 10.1038/s41598-023-37030-2

**Published:** 2023-06-20

**Authors:** Ourania Tachmatzidou, Argiro Vatakis

**Affiliations:** Multisensory and Temporal Processing Laboratory (MultiTimeLab), Department of Psychology, Panteion University of Social and Political Sciences, 136 Syngrou Ave., 17671 Athens, Greece

**Keywords:** Psychology, Human behaviour

## Abstract

In the real world, object arrangement follows a number of rules. Some of the rules pertain to the spatial relations between objects and scenes (i.e., syntactic rules) and others about the contextual relations (i.e., semantic rules). Research has shown that violation of semantic rules influences interval timing with the duration of scenes containing such violations to be overestimated as compared to scenes with no violations. However, no study has yet investigated whether both semantic and syntactic violations can affect timing in the same way. Furthermore, it is unclear whether the effect of scene violations on timing is due to attentional or other cognitive accounts. Using an oddball paradigm and real-world scenes with or without semantic and syntactic violations, we conducted two experiments on whether time dilation will be obtained in the presence of any type of scene violation and the role of attention in any such effect. Our results from Experiment 1 showed that time dilation indeed occurred in the presence of syntactic violations, while time compression was observed for semantic violations. In Experiment 2, we further investigated whether these estimations were driven by attentional accounts, by utilizing a contrast manipulation of the target objects. The results showed that an increased contrast led to duration overestimation for both semantic and syntactic oddballs. Together, our results indicate that scene violations differentially affect timing due to violation processing differences and, moreover, their effect on timing seems to be sensitive to attentional manipulations such as target contrast.

## Introduction

Every object in the world has its own place. This arrangement of items not only is not random but is also characterized by a few rules. These rules are referred to as “syntactic” and “semantic” (as termed in^1^) and indicate the appropriate spatial coordinates of several objects in a visual scene. Syntactic relations refer to the spatial relations of scenes and objects, while semantic relations within an object and a scene are about the probability, size, and position of the given object in the scene. In this latter case, knowing the object’s identity and function is required. A more recent and slightly different interpretation of semantic and syntactic relations among objects and scenes comes from Vo and Wolfe^2^. According to them, semantics are about the object’s fitting into the global context of a scene, while syntactic relations refer to objects being at their expected location in a scene. This notion was further supported by the different neural representations of semantic and syntactic scene violations with the former producing negative deflections in the N300-N400 time window, while the latter positive deflections in the P600^2,3^.

This so-called scene “grammar” is, thus, responsible for our understanding of the visual environment, the recognition of the objects in it, and, moreover, the execution of specific actions depending on the surroundings^4^. It can also assist one in inferring additional information, like the existence of other objects in the scene and their position in it^5^. However, what happens when scene grammar rules are violated, is equally interesting. An early study from Loftus and MacKworth^6^ showed that semantically inconsistent objects in a scene (i.e., an octopus in a farmyard) were fixated faster and for longer times compared to consistent with the scene objects. These findings along with more recent ones^7,8^ suggest that objects that violate scene grammar might attract attention even before becoming fully identified by the observer. That is also known as the pre-attentive pop-out effect^9^. Yet, this effect has not always been reported, with some studies showing that when semantic and syntactic violations are applied, inconsistent objects are identified slower and less accurately than the consistent ones^1,10,11^. In a more recent study, Vo and Henderson^12^ investigated the degree of attentional capture via eye-movements when semantic and syntactic inconsistencies were present in real-world scenes. Their results did not support the pre-attentive pop out account, as they saw that there was no extrafoveal processing of inconsistent objects. However, they found that after fixation, gaze duration for inconsistent objects was longer and that first fixation was even longer for syntactically inconsistent objects compared to semantically inconsistent ones. Thus, suggesting that syntactic and semantic inconsistencies are processed in the fovea area (i.e., the area with the greatest visual acuity that is responsible for object identification) and, moreover, that a prolonged attention allocation is needed (even greater for the syntactic violations). This research points out the need to further investigate not only the perception of those violations as a function of attention, but also their potential differential effects on perception (i.e., semantic versus syntactic).

The effects of scene violations in perception have been investigated in the timing domain (note that we use the terms “timing” and “time perception” interchangeably to refer to the experienced or subjective temporal value that one utilizes to make their judgement; e.g.,^13^). For instance, Clarke and Porubanova^14^ examined the role of semantic knowledge for real-world scenes and objects, hypothesizing that violations of that knowledge would lead to duration overestimations, as a result of the increased neural processing needed for their encoding. They, thus, presented images of real-world scenes with semantic violations or no violations in a duration reproduction task. They also included an attentional manipulation by either drawing or distracting participants’ attention to the violations presented (i.e., participants were asked to report scene-object inconsistencies or the main character’s gender by pressing a key, respectively). Analyses showed that scene duration in the presence of a violation (i.e., semantic) was overestimated as compared to no violations, an effect that was independent of the attentional manipulation used. These findings were interpreted based on the neural energy model of time dilation^15^ that supports that perceived duration correlates positively with the amount of cognitive and neural processing necessary for stimulus encoding (i.e., coding efficiency; cf.^16,17^). This model is an alternative to the idea that subjective time expansion is driven by attentional accounts (e.g.,^18–20^). An example of the latter account can be found in Tse et al.’s^20^ study that showed evidence of time dilation due to the allocation of attention to an unexpected stimulus (cf.^21–23^). Specifically, using an oddball paradigm, where a low-probability stimulus (i.e., odd) is presented in a stream of high-probability stimuli (i.e., standards), the former was perceived as longer compared to the latter stimulation. Such accounts are, also, in line with Schweitzer et al.’s^24^ study, where they utilized contextually-associated (e.g., standard was a pizza and odd a pizza cutter) and non-associated (i.e., standard was a pizza and odd a rubber duck) odds in an oddball paradigm, in which, as expected, both odds were overestimated as compared to standards, yet the contextually-associated odds exceeded the temporal dilation of the non-associated ones. [It must be noted here, however, that the objects in this study were presented in isolation and not in the context of a scene. Thus, this study does not really refer to a scene violation, but rather to a semantic congruence or incongruence of the stimulus stream presented in each trial]. It was argued that the larger dilation occurred due to the attraction of top-down attention to the contextually associated oddballs^24^.

Given the prolonged attentional allocation accounts in the scene grammar violation literature (e.g.,^12^) and the time dilation noted for semantic-like scene violations^14^, one wonders whether any schema violation (i.e., violation of an existing mental construction about the real world; e.g.,^25^) would lead to an expansion of timing and, moreover, whether attention is the main cognitive effect involved in this phenomenon. In the present pre-registered study (see pre-registration here https://doi.org/10.17605/OSF.IO/S3QT5), therefore, we aimed to investigate these issues by conducting two experiments. In Experiment 1, we utilized the oddball paradigm along with naturalistic visual scenes with or without violations, with the violations being of syntactic or semantic form^26^. We hypothesized that in the presence of both semantic and syntactic violations, time dilation would occur due to the prolonged attentional allocation to the violation^14,24^. We, also, expected that this dilation would be greater in the presence of a syntactic as compared to a semantic violation given that the initial encoding of the former requires more time than the latter (cf.^12^). In Experiment 2, we utilized the same setup as in Experiment 1, but we manipulated attention allocation to the target objects of the violation by increasing their contrast relative to their background, an effect known to modulate attention (cf.^27,28^). We hypothesized that time dilation would be observed for both types of violations. Yet, we expected that the effect would be stronger in the increased contrast conditions due to an increased gaze allocation to the target^27^. The results of these two experiments are expected to inform the scene grammar literature in terms of the conflicting accounts of pop-out or prolonged attentional processing and the timing literature in terms of the mechanisms underlying time dilation.

## Experiment 1

### Methods

#### Participants

G*Power^29^ was used to perform an a priori power analysis for a one-sample t-test, comparing participants’ duration estimations with the standard duration. The effect size for this analysis was estimated based on Cohen’s guidelines^30^. This indicated that the best estimate of the true population standardized mean difference was δ = 0.80, meaning that duration estimations will be reported as higher or lower from the standard duration. This effect size estimate was entered into the power analysis with the following input parameters: a (two-sided) = 0.05, power = 0.95. The power analysis results suggested that a *N* = *23* is required in this study to detect a difference between the mean of the estimated durations and the standard duration value.

A similar a priori power analysis was also performed for a repeated measures analysis of variance comparing duration estimation for real-world scenes with violations (i.e., syntactically, semantically) with duration estimations for each type of scene utilized. This indicated that the best estimate of the true population standardized mean difference was δ = 0.40, meaning that the duration estimations will be different among scenes with different violations and type. This effect size estimate was entered into the power analysis with the following input parameters: a = 0.05, power = 0.95. The power analysis results suggested that a *N* = 12 is required in this study to detect a difference between the two conditions with 95% probability.

We recruited twenty-nine participants, assuming that some participants may not follow the experimental instructions or complete the study. All participants were university students (23 female), aged between 19 and 32 years old (mean age = 21) with normal or corrected to normal vision. Participants participated voluntarily for their own interest or the extra credit course opportunities offered in the University through online advertisements and social media posts. When they expressed interest in participating, they were provided with detailed information about the experimental procedure, and they signed the informed consent documentation. This study was approved by the ethics committee of the Panteion University of Social and Political Sciences (protocol number: 33/27-6-2022). All methods included in the present study were performed in accordance with the institution’s relevant guidelines and regulations.

#### Apparatus

The experiment was programmed and run on OpenSesame 3.3^31^. The stimuli were presented on a 21.5-inch FUJITSU Display E22-8 TS Pro computer monitor, set at 1920 × 1080 resolution. The operating system was Windows 10.

#### Stimuli

**Figure 1:**
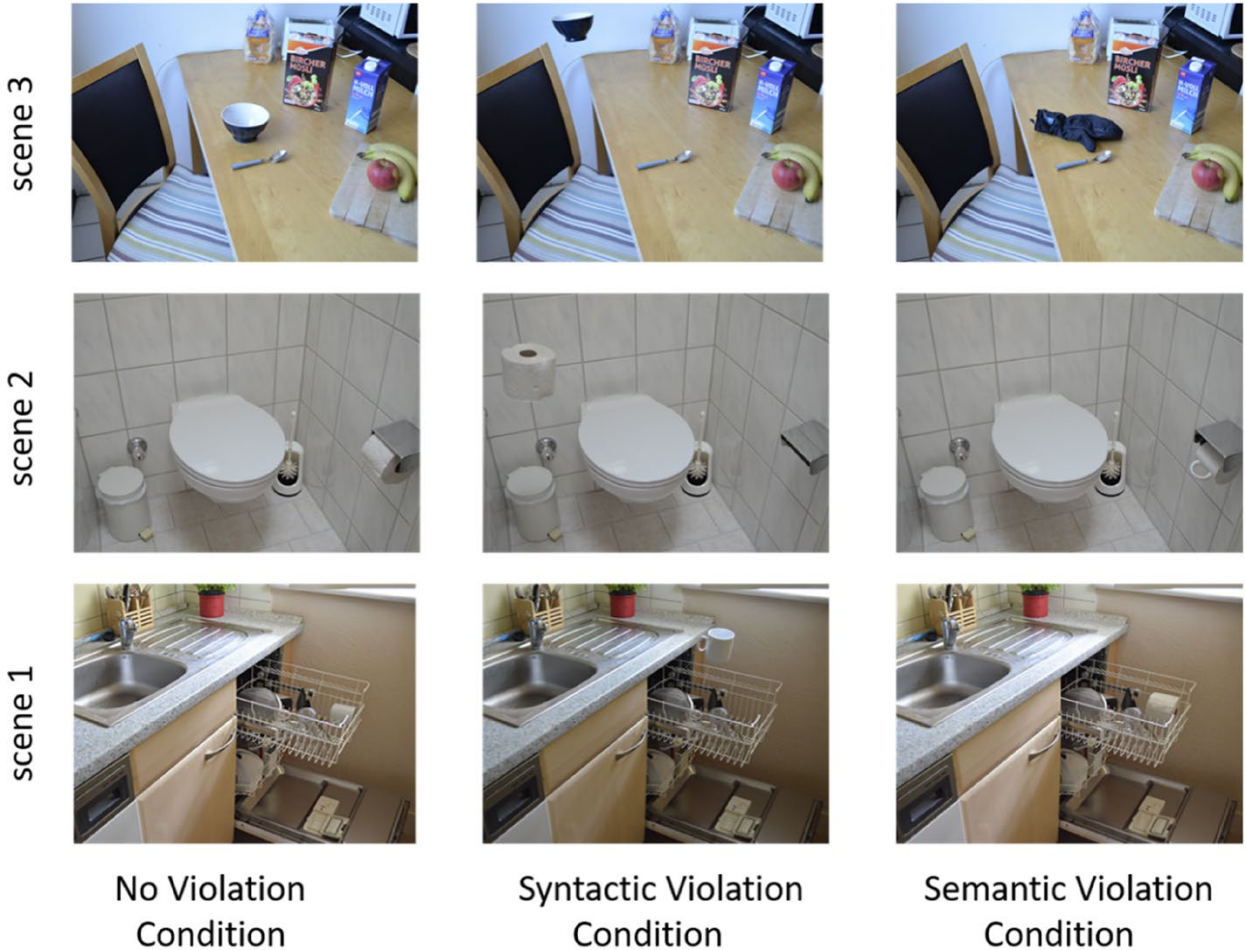
Scenes with and without violations utilized in Experiment 1. The figure depicts the three scenes utilized in the no violation (i.e., standard), semantic violation (e.g., toilet paper in the washing machine), and syntactic violation (e.g., floating cup) condition. These images were adopted from the SCEGRAM database (Öhlschläger and Vo^36^).

Nine color images (i.e., 3 scenes with no violations and their respective scenes with 3 semantic and syntactic violations) with a standard 4:3 aspect ratio and 1.024 × 768-pixel resolution, captured in the real world (i.e., in several different apartments) and containing items that are essential to every household (toilet paper, cup, remote control etc.) were used. These essential items served as the target objects that allowed for the creation of the semantic and syntactic violations. In the semantic violation condition, a semantically inconsistent object was presented in a syntactically consistent location (i.e., a cup in the bathroom). In the syntactic violation condition, a semantically consistent object was presented in a syntactically inconsistent and physically impossible location (i.e., a floating cup in the kitchen; see Fig. 1). All images were taken from the SCEGRAM database, an image-set that contains standardized semantic and syntactic object-scene inconsistencies^26^. We selected the semantic and syntactic item violations to create the semantic and syntactic oddballs, respectively. Images subtended a visual angle of approximately 10° × 11.1° at a viewing distance of approximately 60 cm.

#### Design and procedure

After they sat in a chair in front of the computer monitor, the participants were instructed to fixate at the center of the computer screen. They completed 216 experimental trials, in which they observed 8 repeated presentations of a real-world scene image (i.e., a standard) and one more with a syntactic or semantic violation (i.e., an oddball) randomly presented between the 5^th^ and the 8^th^ position^32^. Images were repeatedly presented at fixation for 500 ms, with ISIs of 300 ms, while the oddballs’ duration varied between 300 and 700 ms, in steps of 50 ms (i.e., 9 different durations). After each trial, participants were asked to report whether the oddball’s duration was longer or shorter than the duration of the images preceding and succeeding it (i.e., standards) by pressing the “,” or the “.” keyboard key (using the same hand, the dominant one), respectively. Both keyboard keys were labelled as “LONG” and “SHORT”, respectively. Before the main experiment, the participants completed 10 practice trials to familiarize themselves with the procedure. For the practice trials, the same three scenes as in the experimental task were used.

## Results and discussion

**Figure 2:**
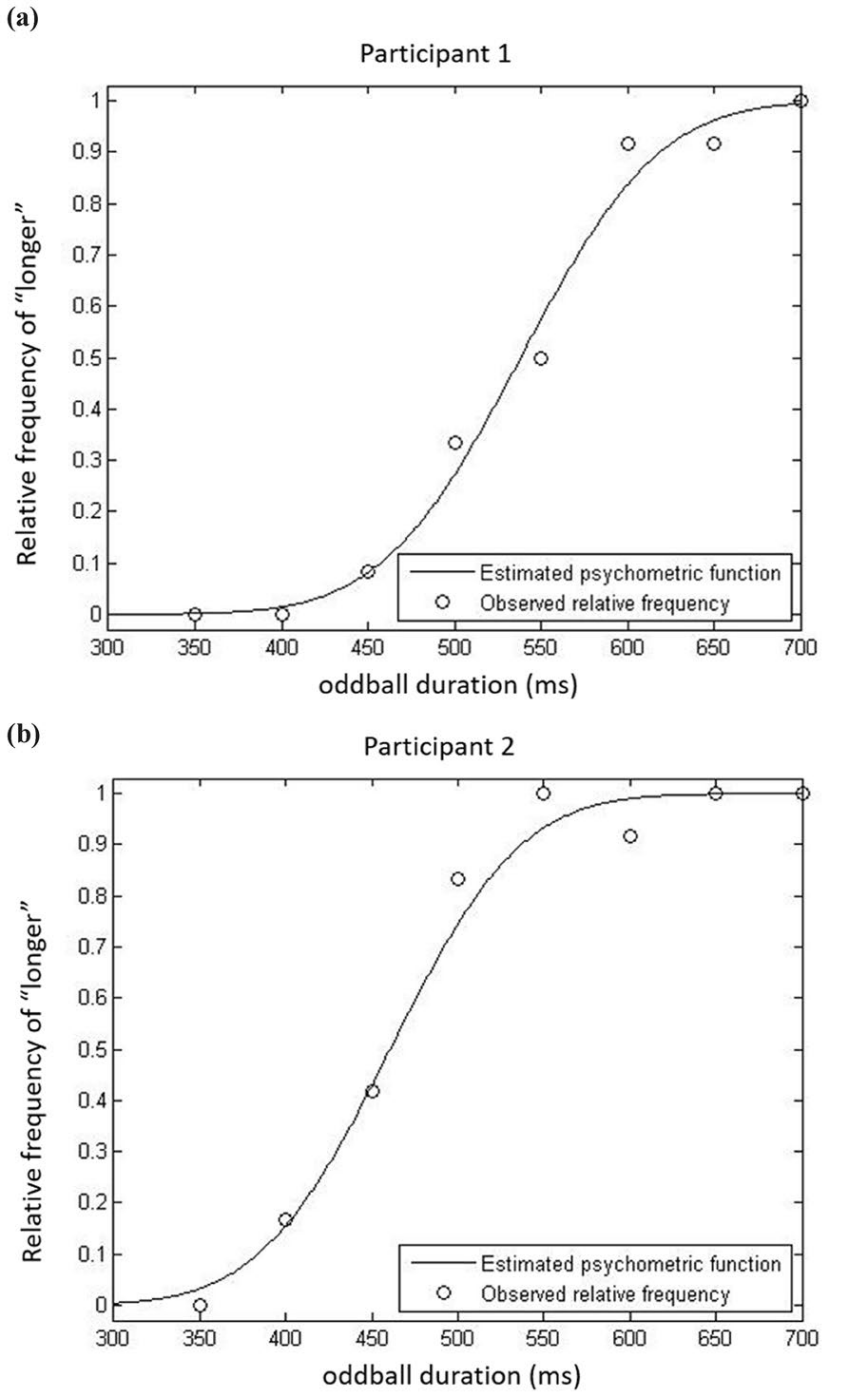
Representative psychometric functions for two participants for the semantic (**a**) and syntactic (**b**) violation conditions in Experiment 1.

**Figure 3:**
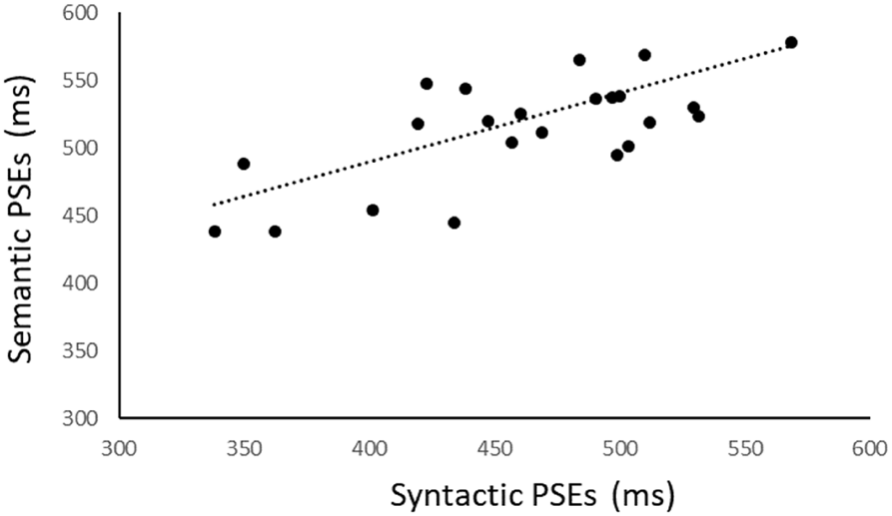
Participants’ Point of Subjective Equality (PSE) of the oddball for the two violation types (i.e., syntactic, semantic) in Experiment 1.

Four participants were removed from the analysis as they failed to follow or understand the given instructions (thus, ending up with a *N* = 25). For all the analyses, Bonferroni-corrected t-tests (where *p* < 0.05 prior to correction) were used for all post-hoc comparisons. When sphericity was violated, Greenhouse-Geisser correction was applied. The psychometric function for each participant was estimated (see Fig. 2), using the MATLAB R2013b software, based on the number of times they reported that the oddball was “longer” than the standard. A psychometric function was fitted to the relative frequencies of “longer” responses per oddball duration level. The cumulative density function (cdf) of a normal distribution was used as the mathematical model for the psychometric function, a modeling approach known as probit analysis (see^33^). The psychometric function was used for estimating the point of subjective equality (PSE; see Fig. 3), which is defined as the oddball’s duration for which the probability of giving the answer “longer” was 50%. That is, an oddball with a longer duration estimate as compared to the standard is being underestimated, while an oddball with a shorter estimate than the standard’s is being overestimated. The PSEs of each participant were used for further statistical analyses.

A one-sample t-test was run to determine whether participants’ PSEs were different to the point of objective equality (POE), that is 500 ms. The analysis showed that PSEs for oddballs with syntactic violations were significantly lower by a mean of 35.9 ms (95% CI [11.9–59.9]) than POE [t(24) = − 3.088, *p* = 0.005]. On the contrary, PSE for oddballs with semantic violations were significantly higher by a mean of 22.3 ms (95% CI [42–2.5]) than POE [t(24) = 2.329, *p* = 0.029]. There was no significant difference of the mean PSE for both oddball types from POE [t(24) = − 0.870, *p* = 0.393].

**Figure 4:**
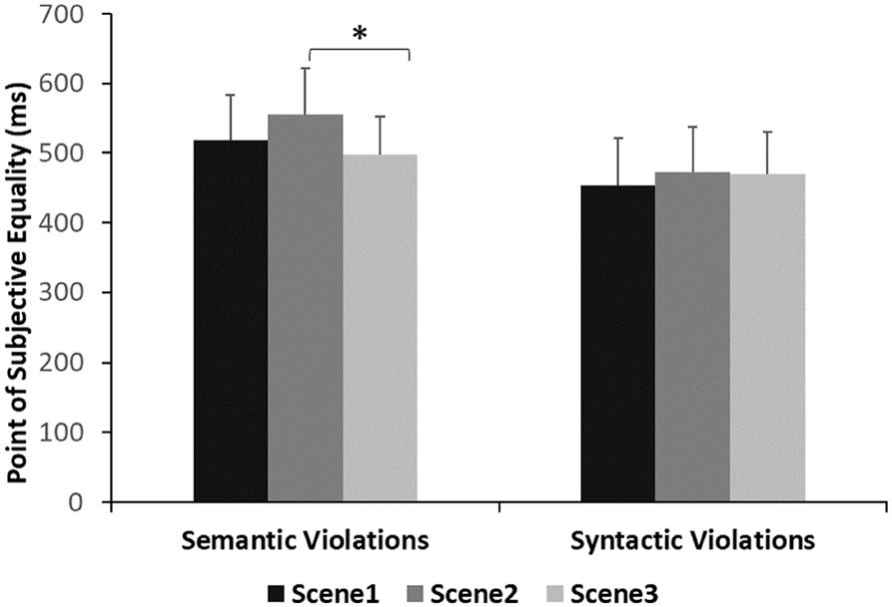
Mean Point of Subjective Equality (PSE) for each Scene per Violation Type (i.e., syntactic, semantic) in Experiment 1. The error bars represent the standard error of the mean. *p < .05.

To further explore the above-mentioned results, data were analyzed using a repeated measures ANOVA between Violation Type (2 levels: syntactic, semantic) and Scene (3 levels: scene 1, scene 2, scene 3). The alpha level was set to 0.05 and the confidence interval to 95%. A significant main effect of Violation Type was obtained [F(1, 24) = 35.82, *p* < 0.001, η_p_^2^ = 0.60], with the oddballs containing semantic violation having a higher PSE (M = 524 ms) as compared to those with the syntactic ones (M = 465.6 ms). A main effect of Scene was also obtained [F(2, 48) = 7.13, *p* = 0.002, η_p_^2^ = 0.23], with scene 2 having higher PSE (M = 514.2 ms) than scene 1 (M = 486.3 ms) and scene 3 (M = 483.8 ms; see Fig. 4). The analysis also revealed an interaction between Violation Type and Scene [F(2, 48) = 6.05, *p* = 0.005, η_p_^2^ = 0.20]. Post hoc analysis with a Bonferroni adjustment revealed that scene 2 (M = 555.5, SD = 13.1) was underestimated at a larger degree as compared to scene 3 (M = 497.5, SD = 10.9; *p* = 0.001), but only in the case of semantic violations.

Overall, the results of Experiment 1 showed that time dilation, indeed, occurs in the presence of syntactic violations, yet, contrary to our predictions, the opposite is true for the semantic violations. This could indicate the existence of differential processing mechanisms for syntactic and semantic violations^2^. Moreover, we observed that in the presence of semantic violations, one of the scenes (i.e., scene 2; see Fig. 1) utilized was significantly underestimated. That could possibly mean that apart from the semantic violation itself, additional scene properties (such as brightness^34^;) might affect the scenes’ percept. In Experiment 2, we extended our research by questioning whether the timing modulations observed were influenced by attentional accounts (inspired by^14^).

## Experiment 2

### Methods

#### Participants

G*Power^29^ was calculated to perform an a priori power analysis for a repeated measures analysis of variance comparing duration estimation for real-world scenes with semantic and syntactic violations with no contrast manipulations of the target objects with the same scenes but with increased contrast of the target objects. The effect size for this analysis was estimated based on Cohen’s^30^ guidelines. This indicated that the best estimate of the true population standardized mean difference was δ = 0.40, meaning that the duration estimations will be different among scenes with different violations and target objects’ contrast. This effect size estimate was entered into the power analysis with the following input parameters: a = 0.05, power = 0.95. The power analysis results suggested that a *N* = 15 was required in this study to detect a difference between the two conditions with 95% probability.

We recruited 19 participants, assuming that not all will follow the experimental instructions or complete the experiment. All participants were university students (10 females), aged between 21 and 44 years old (mean age = 26) with normal or corrected to normal vision.

#### Apparatus and stimuli

These are in line with Exp. 1, with only a few exceptions. Two scenes from the SCEGRAM database were utilized here as visual stimulation^26^. These scenes were scene’s 1 and 2 from Exp. 1 (see Fig. 1) to keep the duration of the experiment short. To manipulate attention, the oddballs were modified by using Adobe Photoshop 2020 Software^35^. To draw the participant’s attention to the target object, the object’s contrast was increased by dragging the contrast slider to + 100 (cf.^28,36^). This manipulation led to an approximately 20% difference in intensity (i.e., mean gray value) of the pixels in the region of interest (i.e., target objects) for the manipulated stimuli, as it was afterwards measured with ImageJ software. However, the above attentional manipulation should be verified in future research.

#### Design and procedure

These were also in line with Exp 1, with a few exceptions. The participants ran 288 experimental trials, in which they observed 8 repeated presentations of the standard stimuli and one with an oddball, manipulated in terms of contrast to either draw more attention to the target object or not (i.e., contrast vs. no contrast manipulation of the target object, respectively).

## Results and discussion

**Figure 5:**
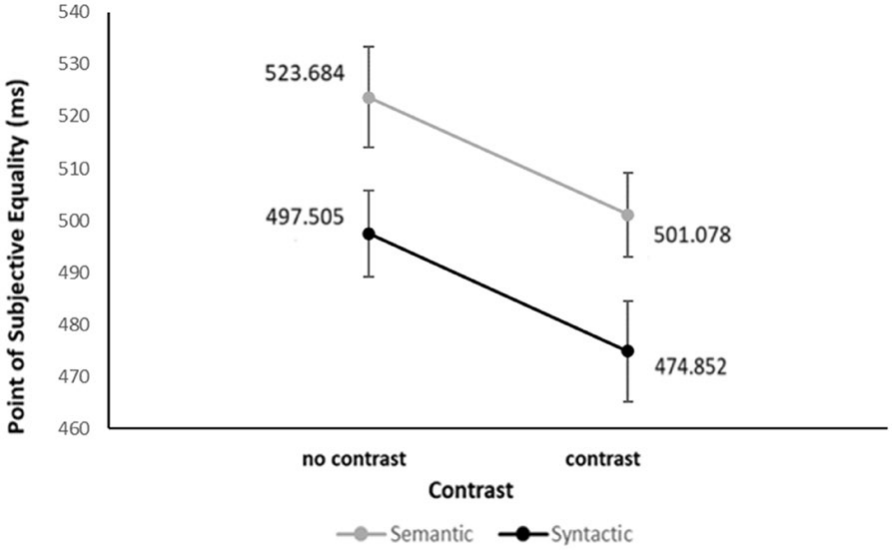
Mean Point of Subjective Equality (PSE) for each Violation Type (i.e., syntactic, semantic) per Contrast (i.e., contrast, no contrast manipulation) condition in Experiment 2.

Three participants were removed from the analysis as they failed to follow directions or did not understand the given instructions (thus, ending up with a *N* = 16). A repeated measures ANOVA of Violation Type (2 levels: syntactic, semantic), Contrast (2 levels: contrast or no contrast manipulation), and Scene (2 levels: scene 1, scene 2) was conducted. The alpha level was set to 0.05 and the confidence interval to 95%. A significant main effect of Violation Type was obtained [F(1, 15) = 18.24, *p* = 0.001, η_p_^2^ = 0.549], with the oddballs containing semantic violations having a higher PSE (M = 512.38 ms) as compared to those with syntactic ones (M = 486.2 ms). A main effect of Scene was also obtained [F (1, 15) = 18.89, *p* = 0.001, η_p_^2^ = 0.557], with scene 2 having a higher PSE (M = 517.1 ms) than scene 1 (M = 481.5 ms). Moreover, a significant main effect of Contrast was obtained [F (1, 15) = 13.65, *p* = 0.002, η_p_^2^ = 0.476], with scenes containing contrast manipulations having a lower PSE (M = 487.97 ms) as compared to those with no contrast manipulation (M = 510.6 ms; see Fig. 5). The interactions between Violation Type and Scene [F(1, 15) = 0.300, *p* = 0.592, η_p_^2^ = 0.02], Violation Type and Contrast [F(1, 15) = 0.000, *p* = 0.996, η_p_^2^ = 0.00], Contrast and Scene [F(1, 15) = 1.163, *p* = 0.298, η_p_^2^ = 0.07], and Violation Type, Contrast, and Scene [F(1, 15) = 0.878, *p* = 0.363, η_p_^2^ = 0.055] did not reach significance. Overall, the results of Exp. 2 showed that by focusing the participants’ attention more to the violation lead to higher duration estimates. This was observed for all cases presented in the experiment, even for the highly underestimated semantically violated scenes (it must be noted, however, that no significant Violation Type by Contrast or triple interaction were obtained). Such findings indicate that this time dilation effect is probably driven by attentional accounts (e.g.,^18–20^).

### General discussion

In the present study, we investigated the effect of semantic and syntactic violations on time perception, by utilizing the oddball paradigm along with naturalistic visual scenes (Experiment 1), as well as the role of attention on these estimates by manipulating target objects’ contrast (Experiment 2). We tested the hypothesis that both semantic and syntactic scene violations would lead to time dilation, which was expected to be more pronounced for the syntactic violation types. Moreover, we hypothesized that this effect would be stronger in the increased contrast conditions due to an increased gaze allocation to the target. Our results showed that time dilation, indeed, occurs in the presence of syntactic violations, yet, contrary to our predictions, the opposite applies for the semantic violations of a scene (Experiment 1). Furthermore, it appears that these time distortions are, indeed, affected by attentional allocation, as an increase in target objects’ contrast led to an increased duration percept for both semantic and syntactic scene violations (Experiment 2).

Previous studies employing the oddball paradigm have reported that the appearance of an unexpected visual^15,20,32,37–39^ or auditory (cf.^32,40,41^) stimulus, in a stream of identical ones, can affect subjective duration judgments and lead to duration overestimations of the odd stimulation. Given that, to our knowledge, no studies on timing had tested whether more complex stimuli (i.e., natural scenes) could induce similar effects in an oddball setting (see also^42–44^ for the use of more complex stimuli in variations of the oddball paradigm yet not in terms of time perception), we reasoned that our findings could further expand the literature around the oddball effect, as well as provide evidence on the role of attention on intervals’ duration estimates.

And indeed, our findings expanded current knowledge by showing that odds in complex scenes are not always overestimated. Instead, the timing of the odds varies as a function of the scene violation present (i.e., syntactic vs. semantic). We argue that the time estimation difference we obtained between the two violation types in Experiment 1 (and replicated in Experiment 2) might indicate the presence of a differential processing mechanism for syntactic and semantic scene violations. A review of the existing literature of scene perception, indicates that there is an influence of scene context on object perception and vice versa (e.g.,^45–47^). For example, Davenport and Potter^45^ studied the effects of scene consistency on perception in a series of experiments. They presented real-world photographs, including foreground objects semantically consistent or inconsistent with their background and asked their participants to identify these objects, their background or both, after seeing a photograph briefly followed by a mask. Their findings showed that when objects appeared in a semantically inconsistent background, they were identified less accurately than in a consistent one. The same applied for the background and both object-background identification. These results clearly demonstrate that in the presence of semantic violations, scene perception is impaired. Moreover, according to Joubert et al.’s ^48^ study, semantically incongruent objects in a scene not only affect perceptual accuracy but also reaction time. In a go/no go task, participants in Joubert et al.’s study were asked to categorize scenes shown briefly as either natural or man-made environments. Analyses of the data showed that it took participants significantly more time to categorize the scenes that included objects incongruent with the context (i.e., man-made objects on a natural background) as compared to the scenes with congruent objects. By combining functional imaging and behavioral data, Rémy et al.’s^47^ aimed not only to investigate participants’ performance in categorizing real-world scenes with semantic violations, but also to detect the neural corelates underlying this process. Their behavioral data analyses replicated the impaired performance previously reported in the processing of scenes with semantic violations, and, moreover, the combined analyses with the fMRI findings revealed an increased activation in specific brain areas (i.e., right anterior PHC, right frontal cortex, posterior part of PHC) in response to semantic violations.

The above-mentioned findings support the idea that the detection of semantic violations as well as the processing of scenes and objects with such violations appears to be a demanding task. This could provide an explanation for the results described in the present study. We support that the appearance of semantic violations in scenes distracted participants from the timing task -by allocating attention to the violation per se- and as a result led to significant underestimations of these intervals. This explanation is in line with the theory behind the interference effect^49^, a robust finding among the timing literature^49–52^. According to this effect, when a temporal task co-occurs with a demanding non-temporal one, the latter acts as a distractor and disrupts the performance of the former one, by holding attention away from time. As a result, the duration estimates of the intervals presented appear to be shorter and possibly less accurate and more inconsistent (cf.^53^). The explanation of this effect is in line with the internal clock timing models^54–56^ that suggest that time perception is manageable due to the existence of an internal clock-like mechanism. An internal clock is a (hypothetical) mechanism, containing a neural pacemaker that produces pulses. The exact number of pulses that are related to a physical time interval are recorded by another part of this clock, the counter, and then the results are stored in a so-called store^55^. By distracting attention away from the timing task, less pulses are being counted and, thus, interval durations are perceived as shorter^57^.

So far, it seems that the processing of scenes with semantic violations possibly leads to shorter duration estimations due to their distractive effect on the timing task. However, according to the results of Experiment 1, this might not be the case for syntactic violations, where we observed significant duration overestimation of the scenes that included such violations. In their research, Gronau et al.’s^46^ also highlighted the difference between semantic and syntactic violations. They investigated the relation between semantic (i.e., information about the object’s identities that are most likely to appear within a specific visual setting) and spatial (i.e., information about the locations within a visual setting that is most likely for an object to appear) contextual knowledge in a behavioral and neural level. Participants performed a priming task, in which the prime stimulus was a real-world object appearing at the center of the screen and the target stimulus was either a semantically consistent or inconsistent one, appearing at an upper or lower location of the scene. The task was to answer whether the target object was semantically consistent (i.e., “real object”) or inconsistent (i.e., “nonsense object”) with the scene. The behavioral data analyses showed that responding for semantically inconsistent targets was significantly slower compared to semantically consistent ones. This was not the case for the spatially inconsistent targets. Although further investigation is necessary, it appears that the processing of syntactic violations is, indeed, different from the processing of semantic violations, thus explaining the differences we observed in our experiments. We support the idea that while semantic violation processing and detection might have been capable of drawing attention away from the timing task (i.e., leading to duration underestimation), syntactic violations attracted observers’ attention and, thus, the replication of the temporal oddball effect as hypothesized. This idea is supported by Vo and Wolfe’s^2^ neurological findings that indicate an increased post-identification processing of semantic violations, while no such findings were noted for extreme syntactic violations (i.e., hovering objects; cf.^58,59^). However, in contrast with our results, there are also evidence of time overestimation of semantically violated scenes (see^14^). At this point it should be noted that in Clarke and Porubanova’s^14^ study, a different time estimation method was utilized (i.e., a reproduction task). It is a common finding that shorter time intervals are more likely to be overestimated as compared to longer intervals^60^ and, on top of that, motor factors might also influence an intervals’ reproduction (e.g.,^61,62^). Nevertheless, further investigation of the differences among the two violation types is needed to clarify the mechanisms behind their processing and how that can influence time perception.

Another interesting finding of our study is the interaction observed in Experiment 1 between Violation Type and Scene. We found that the duration of scene 2 was significantly underestimated compared to scene 3 (see Fig. 1), in the presence of semantic violations only. This could indicate that additional to the violation itself, other properties of these scenes might influence their processing such as the color or the location of the target objects. More specifically, it has been found that the detection of a target might take longer when this has a similar color with the background that is presented against^63^. Moreover, the possibility that the location of the objects in a scene might influence their perception was investigated by Spotorno and Faure^64^. By using a change-detection task, they briefly presented pairs of colored drawings, depicting real-world situations. Participants had to report whether each pair was identical or not. The changes included the addition of an object either to the left or to the right visual hemifield. The results showed that left visual hemifield has an advantage for detecting changes in scenes. There is also evidence in the visual search literature that targets appearing near fixation (i.e., the target object in scene 3 in our study) are detected more quickly compared to those in periphery^65,66^. Considering these findings, it is important to further investigate the influence of the interaction between contextual and perceptual scene properties on time estimation.

Until now, it was unclear whether the time distortions we observed in Experiment 1 were driven by attention. Therefore, in Experiment 2, we used an increased target objects’ contrast to manipulate attentional allocation. The possibility that contrast can affect attentional allocation in natural scenes was investigated by ‘t Hart et al.’s^27.^ In their study, they used natural scenes in which they modified luminance contrast of specific objects. In one of their experimental setups, they asked participants to freely view the briefly presented scenes, while they were recording their eye movements. In their second experimental setup, participants viewed a stream of scenes (including the above-mentioned manipulated ones) and reported whether they detected or not a target object. The results showed that by increasing objects’ contrast, relative to their background, both fixations to these objects and their detection increased (cf.^67–71^). In the timing literature, the increase of the subjective duration of an attended object is a quite robust effect and it has been tested with a variety of experimental paradigms (see^18^, for a review). In the present study, by increasing target objects’ luminance contrast, we observed an increase in the oddballs’ duration estimates for both semantic and syntactic violations and, therefore, we support that these results stem from attentional factors (e.g.,^18–20^). Thus, our study adds on the literature supporting the prolonged attentional processing of scene violations^1,10–12^.

However, it is remarkable that the effect of semantic violations on duration underestimation was maintained despite the increased contrast manipulation, thus indicating a high interference effect of the violation type on timing. The idea that there might be an interaction between the perceptual salience of objects (i.e., brightness, color, orientation; cf.^72^) and their semantic relevance during scene perception was investigated by Spotorno et al.’s^34^. They used a one-shot change detection task and colored drawings of daily-life events as stimuli and showed that semantically consistent objects, as well as objects with higher salience were faster and more accurately detected whether they were added to or deleted from a scene. They supported that visual attention is primarily guided from perceptual properties of objects and semantic properties have a supplementary effect (cf.^64,69,73^). Therefore, our results further support the high influence of semantic congruency on object detectability (i.e., easy of being detected).

In conclusion, by utilizing an oddball paradigm, we showed that both semantic and syntactic scene violations influence time perception, yet in a different way. For the syntactic violations, we observed perceptual effects similar to those observed in most of the temporal oddball effect literature, where perceived time expands in their presence. On the other hand, for the semantic violations, we obtained a reversed pattern on temporal oddballs with perceived time contracting. These findings indicate that by moving to more naturalistic contexts, timing and its interaction with attention -wherever this may be allocated to- might work differently and so do well-established and robust effects in basic literature. To our knowledge, our study is the first to obtain such findings, so further research on timing under naturalistic settings can further clarify this area. Moreover, our findings expand current knowledge on the perception of naturalistic scenes and how scene properties can affect this percept. Lastly, our work adds to the literature demonstrating that perceived duration can be influenced by attention, as noted here through the increased duration estimates when manipulating attention allocation via contrast changes.

## Data Availability

The data and materials for all experiments are available at https://osf.io/ub8wg/.

## References

[1] Biederman I, Mezzanotte RJ, Rabinowitz JC (1982). Scene perception: Detecting and judging objects undergoing relational violations. Cogn. Psychol..

[2] Võ ML-H, Wolfe JM (2013). Differential electrophysiological signatures of semantic and syntactic scene processing. Psychol. Sci..

[3] McCormick C, Maguire EA (2021). The distinct and overlapping brain networks supporting semantic and spatial constructive scene processing. Neuropsychologia.

[4] Võ ML (2021). The meaning and structure of scenes. Vis. Res..

[5] Bar M (2004). Visual objects in context. Nat. Rev. Neurosci..

[6] Loftus GR, Mackworth NH (1978). Cognitive determinants of fixation location during picture viewing. J. Exp. Psychol. Hum. Percept. Perform..

[7] Bonitz VS, Gordon RD (2008). Attention to smoking-related and incongruous objects during scene viewing. Acta Physiol..

[8] Gordon RD (2004). Attentional allocation during the perception of scenes. J. Exp. Psychol. Hum. Percept. Perform..

[9] Wu CC, Wick FA, Pomplun M (2014). Guidance of visual attention by semantic information in real-world scenes. Front. Psychol..

[10] Davenport JL, Potter MC (2004). Scene consistency in object and background perception. Psychol. Sci..

[11] Kelley TA, Chun MM, Chua KP (2003). Effects of scene inversion on change detection of targets matched for visual salience. J. Vis..

[12] Võ ML, Henderson JM (2009). Does gravity matter? Effects of semantic and syntactic inconsistencies on the allocation of attention during scene perception. J. Vis..

[13] Allan LG (1979). The perception of time. Percept. Psychophys..

[14] Clarke J, Porubanova M (2020). Scene and object violations cause subjective time dilation. Timing Time Percept..

[15] Eagleman DM, Pariyadath V (2009). Is subjective duration a signature of coding efficiency?. Philos. Trans. R. Soc. Lond. Ser. B Biol. Sci..

[16] Grill-Spector K, Henson R, Martin A (2006). Repetition and the brain: Neural models of stimulus-specific effects. Trends Cogn. Sci..

[17] Todorovic A, de Lange FP (2012). Repetition suppression and expectation suppression are dissociable in time in early auditory evoked fields. J. Neurosci..

[18] Matthews WJ, Meck WH (2016). Temporal cognition: Connecting subjective time to perception, attention, and memory. Psychol. Bull..

[19] Rose D, Summers J (1995). Duration illusions in a train of visual stimuli. Perception.

[20] Tse PU, Intriligator J, Rivest J, Cavanagh P (2004). Attention and the subjective expansion of time. Percept. Psychophys..

[21] Birngruber T, Schröter H, Schütt E, Ulrich R (2018). Stimulus expectation prolongs rather than shortens perceived duration: Evidence from self-generated expectations. J. Exp. Psychol. Hum. Percept. Perform..

[22] Block RA, Gruber RP (2014). Time perception, attention, and memory: A selective review. Acta Physiol. (Oxf.).

[23] New JJ, Scholl BJ (2009). Subjective time dilation: Spatially local, object-based, or a global visual experience?. J. Vis..

[24] Schweitzer R, Trapp S, Bar M (2017). Associated information increases subjective perception of duration. Perception.

[25] Baldassano C, Hasson U, Norman KA (2018). Representation of real-world event schemas during narrative perception. J. Neurosci. Off. J. Soc. Neurosci..

[26] Öhlschläger S, Võ ML (2017). SCEGRAM: An image database for semantic and syntactic inconsistencies in scenes. Behav. Res. Methods.

[27] t’Hart BM, Schmidt HC, Klein-Harmeyer I, Einhäuser W (2013). Attention in natural scenes: Contrast affects rapid visual processing and fixations alike. Philos. Trans. R. Soc. Lond. Ser. B Biol. Sci..

[28] Camgöz N, Yener C, Güvenç D (2004). Effects of hue, saturation, and brightness: Part 2: Attention. Color Res. Appl..

[29] Faul F, Erdfelder E, Buchner A, Lang A-G (2009). Statistical power analyses using G*Power 3.1: Tests for correlation and regression analyses. Behav. Res. Methods.

[30] Cohen J (1988). Statistical Power Analysis for the Behavioral Sciences.

[31] Mathôt S, Schreij D, Theeuwes J (2012). OpenSesame: An open-source, graphical experiment builder for the social sciences. Behav. Res. Methods.

[32] Pariyadath V, Eagleman D (2007). The effect of predictability on subjective duration. PLoS One.

[33] Bausenhart KM, Di Luca M, Ulrich R, Vatakis A, Balci F, Di Luca M, Correa A (2018). Assessing duration discrimination: Psychophysical methods and psychometric function analysis. Timing and Time Perception Procedures, Measures, and Applications.

[34] Spotorno S, Tatler BW, Faure S (2013). Semantic consistency versus perceptual salience in visual scenes: Findings from change detection. Acta Physiol..

[35] Adobe Inc. Adobe Photoshop [Internet]. 2020. Available from: https://www.adobe.com/products/photoshop.html

[36] Camgöz N, Yener C, Güvenç D (2002). Effects of hue, saturation, and brightness on preference. Color Res. Appl..

[37] Pariyadath V, Eagleman DM (2008). Brief subjective durations contract with repetition. J. Vis..

[38] Schindel R, Rowlands J, Arnold DH (2011). The oddball effect: Perceived duration and predictive coding. J. Vis..

[39] Birngruber T, Schröter H, Ulrich R (2015). The influence of stimulus repetition on duration judgments with simple stimuli. Front. Psychol..

[40] Birngruber T, Schröter H, Ulrich R (2014). Duration perception of visual and auditory oddball stimuli: Does judgment task modulate the temporal oddball effect?. Atten. Percept. Psychophys..

[41] Chen KM, Yeh SL (2009). Asymmetric cross-modal effects in time perception. Acta Physiol..

[42] File D, Petro B, Gaál ZA, Csikós N, Czigler I (2022). Automatic change detection: Mismatch negativity and the now-classic Rensink, O'Reagan, and Clark (1997) stimuli. Front. Psychol..

[43] Zaehle T, Bauch EM, Hinrichs H, Schmitt FC, Voges J, Heinze HJ, Bunzeck N (2013). Nucleus accumbens activity dissociates different forms of salience: Evidence from human intracranial recordings. J. Neurosci. Off. J. Soc. Neurosci..

[44] Brandman T, Peelen MV (2017). Interaction between scene and object processing revealed by human fMRI and MEG decoding. J. Neurosci. Off. J. Soc. Neurosci..

[45] Davenport JL (2007). Consistency effects between objects in scenes. Mem. Cognit..

[46] Gronau N, Neta M, Bar M (2008). Integrated contextual representation for objects' identities and their locations. J. Cogn. Neurosci..

[47] Rémy F, Vayssière N, Pins D, Boucart M, Fabre-Thorpe M (2014). Incongruent object/context relationships in visual scenes: Where are they processed in the brain?. Brain Cogn..

[48] Joubert OR, Rousselet GA, Fize D, Fabre-Thorpe M (2007). Processing scene context: Fast categorization and object interference. Vis. Res..

[49] Brown SW (1997). Attentional resources in timing: Interference effects in concurrent temporal and nontemporal working memory tasks. Percept. Psychophys..

[50] Botto M, Palladino P (2016). Time and interference: Effects on working memory. Br. J. Psychol..

[51] Brown SW (2006). Timing and executive function: Bidirectional interference between concurrent temporal production and randomization tasks. Mem. Cognit..

[52] Rattat AC, Fortin C (2011). Modulating the interference effect in timing with varying stimulus onset asynchrony. Can. J. Exp. Psychol..

[53] Brown SW, Collier SA, Night JC (2013). Timing and executive resources: Dual-task interference patterns between temporal production and shifting, updating, and inhibition tasks. J. Exp. Psychol. Hum. Percept. Perform..

[54] Gibbon J (1977). Scalar expectancy theory and Weber's law in animal timing. Psychol. Rev..

[55] Treisman M (1963). Temporal discrimination and the indifference interval: Implications for a model of the "internal clock". Psychol. Monogr. Gen. Appl..

[56] Zakay D, Block RA, Pastor MA, Artieda J (1996). The role of attention in time estimation processes. Time, Internal Clocks and Movement.

[57] Brown SW, Grodin S (2008). Time and attention: Review of the literature. Psychology of Time.

[58] Ganis G, Kutas M (2003). An electrophysiological study of scene effects on object identification. Cogn. Brain Res..

[59] Mudrik L, Lamy D, Deouell LY (2010). ERP evidence for context congruity effects during simultaneous object-scene processing. Neuropsychologia.

[60] Lejeune H, Wearden JH (2009). Vierordt's The experimental study of the time sense (1868) and its legacy [Review of the book&nbsp;The experimental study of the time sense, by K. Vierordt]. Eur. J. Cogn. Psychol..

[61] Droit-Volet S (2010). Stop using time reproduction tasks in a comparative perspective without further analyses of the role of the motor response: The example of children. Eur. J. Cogn. Psychol..

[62] Indraccolo A, Spence C, Vatakis A, Harrar V (2016). Combined effects of motor response, sensory modality, and stimulus intensity on temporal reproduction. Exp. Brain Res..

[63] Farmer EW, Taylor RM (1980). Visual search through color displays: Effects of target-background similarity and background uniformity. Percept. Psychophys..

[64] Spotorno S, Faure S (2011). Change detection in complex scenes: Hemispheric contribution and the role of perceptual and semantic factors. Perception.

[65] Carrasco M, Evert DL, Chang I, Katz SM (1995). The eccentricity effect: Target eccentricity affects performance on conjunction searches. Percept. Psychophys..

[66] Wolfe JM, O’Neill P, Bennett SC (1998). Why are there eccentricity effects in visual search? Visual and attentional hypotheses. Percept. Psychophys..

[67] Anderson NC, Ort E, Kruijne W, Meeter M, Donk M (2015). It depends on when you look at it: Salience influences eye movements in natural scene viewing and search early in time. J. Vis..

[68] Engmann S, ‘t Hart BM, Sieren T, Onat S, König P, Einhäuser W (2009). Saliency on a natural scene background: Effects of color and luminance contrast add linearly. Atten. Percept. Psychophys..

[69] ‘t Hart BM, Schmidt HC, Roth C, Einhäuser W (2013). Fixations on objects in natural scenes: Dissociating importance from salience. Front. Psychol..

[70] Parkhurst D, Law K, Niebur E (2002). Modeling the role of salience in the allocation of overt visual attention. Vis. Res..

[71] Parkhurst DJ, Niebur E (2004). Texture contrast attracts overt visual attention in natural scenes. Eur. J. Neurosci..

[72] Itti L, Koch C (2000). A saliency-based search mechanism for overt and covert shifts of visual attention. Vis. Res..

[73] Nuthmann A, Clayden AC, Fisher RB (2021). The effect of target salience and size in visual search within naturalistic scenes under degraded vision. J. Vis..

